# An Another Protocol to Make Sulfur Embedded Ultrathin Sections of Extraterrestrial Small Samples

**DOI:** 10.3390/life10080135

**Published:** 2020-08-05

**Authors:** Takaaki Noguchi, Minako Takase, Rikako Matsumoto, Yoko Kebukawa, Hiroki Suga, Masashi Kondo, Yoshio Takahashi, Yasuo Takeichi, Hikaru Yabuta

**Affiliations:** 1 Faculty of Arts and Science, Kyushu University, 744 Motooka, Nishi-ku, Fukuoka 819-0395, Japan; 2 Department of Earth and Planetary Science, Kyushu University, 744 Motooka, Nishi-ku, Fukuoka 819-0395, Japan; takase.minako.231@s.kyushu-u.ac.jp (M.T.); rikako.matsumoto.417@gmail.com (R.M.); 3 Faculty of Engineering, Yokohama National University, 79-5 Tokiwadai, Hodogaya-ku, Yokohama 240-8501, Japan; kebukawa@ynu.ac.jp; 4 Department of Earth and Planetary Science, The University of Tokyo, Hongo, Bunkyo-ku, Tokyo 113-0033, Japan; hiroki-suga@spring8.or.jp (H.S.); ytakaha@eps.s.u-tokyo.ac.jp (Y.T.); 5 Instrumental Analysis Center, Yokohama National University, 79-5 Tokiwadai, Hodogaya-ku, Yokohama 240-8501, Japan; kondo-masashi-fj@ynu.ac.jp; 6 Institute of Materials Structure Science, High-Energy Accelerator Research Organization, 1-1 Oho, Tsukuba, Ibaraki 305-0801, Japan; yasuo.takeichi@kek.jp; 7 Department of Earth and Planetary Systems Science, Graduate School of Science, Hiroshima University, 1-3-1 Kagamiyama, Higashi-Hiroshima, Hiroshima 739-8526, Japan; hyabuta@hiroshima-u.ac.jp

**Keywords:** Murchison, IOM, sulfur embedding, ultramicrotomy, STXM–XANES

## Abstract

Another protocol to make sulfur embedded ultrathin sections was developed for STXM–XANES, AFM–IR and TEM analyses of organic materials in small extraterrestrial samples. Polymerized liquid sulfur—instead of low-viscosity liquid sulfur—is the embedding media in this protocol. Due to high viscosity of the polymerized sulfur, the embedded samples stay near the surface of polymerized liquid sulfur, which facilitates trimming of glassy sulfur and ultramicrotomy of tiny embedded samples. In addition, well-continued ribbons of ultramicrotomed sections can be obtained, which are suitable for the above mentioned analyses. Because there is no remarkable difference in Carbon XANES spectra of Murchison IOM prepared by this protocol and by the conventional protocol, this protocol gives another alternative to prepare sulfur embedded ultramicrotomed sections.

## 1. Introduction

Sulfur embedding ultramicrotomy was originally devised to measure energy-loss near-edge structure (ELNES) of light elements such as carbon, oxygen and nitrogen in organic material included in interplanetary dust particles (IDPs) by using electron energy loss spectrometer (EELS) equipped on transmission electron microscope (TEM) [1]. More recently, S embedding ultramicrotomed sections have been used for X-ray absorption near-edge spectroscopy of light elements such as carbon, oxygen and nitrogen in organic material in IDPs, meteorites, the returned Comet 81P/Wild 2 dust particles and ancient terrestrial samples by using scanning transmission X-ray microscopy (STXM–XANES) at synchrotron facilities e.g., [2,3,4,5,6,7,8], which has enabled in situ analysis of submicron-sized extraterrestrial organic materials. Focused ion beam (FIB) processing becomes more common to prepare thin samples for the light elements STXM–XANES analysis of organic materials in extraterrestrial samples e.g., [9,10,11,12,13,14]. However, sulfur embedding ultramicrotomy has an advantage to make ultrathin (~100-nm-thick) foil samples of highly porous and fragile IDPs and AMMs if preservation of their original textures is required. In the end of 2020, samples of the asteroid (162173) Ryugu will be returned to the Earth by the Hayabusa 2 spacecraft. If the asteroid samples are porous and fragile, S embedding ultramicrotomy may play an important role for the sample preparation of thin foils for XANES and EELS analyses. Here we present a new protocol of S embedding ultramicrotomy, which is an easier way to obtain well-continued homogeneously thin ribbons of S embedding ultrathin sections of small samples than the previous methods.

## 2. Materials and Methods

Insoluble organic matter (IOM) extracted from Murchison CM chondrite was used for this study. We analyzed sulfur embedded ultrathin samples prepared by the conventional sample preparation protocol in addition to the ultrathin samples prepared by this protocol. The procedure of the conventional protocol was reported in [8].

Carbon X-ray absorption near-edge structure (C-XANES) analyses were performed using the scanning transmission X-ray microscopes (STXM) at BL-19A of the Photon Factory, High Energy Accelerator Research Organization (KEK) [15]. The C *K*-edge–XANES spectra were acquired with the energy step sizes of 0.1 eV in 283–295.5 eV region, 0.5 eV in 280–283 eV and 295.5–301.0-eV regions and 1 eV in 301–320-eV region, with a dwell time of 5 or 6 ms and 0.2-μm steps per pixel at each region of interest (7 × 6 μm^2^ up to 10 × 10 μm^2^). Three-point smoothing was applied to the raw C-XANES spectra to reduce noises and then normalized with the intensity at 296 eV after subtracting baselines fitted by liner approximations at 280–283 eV.

## 3. Protocol

**Figure 1:**
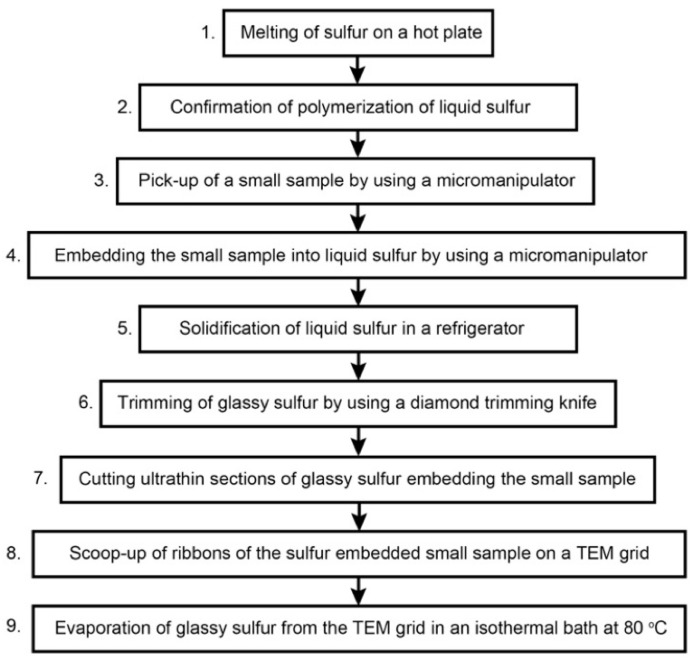
Flow chart of the protocol for sulfur embedding ultramicrotomy developed in this study.

**Figure 2:**
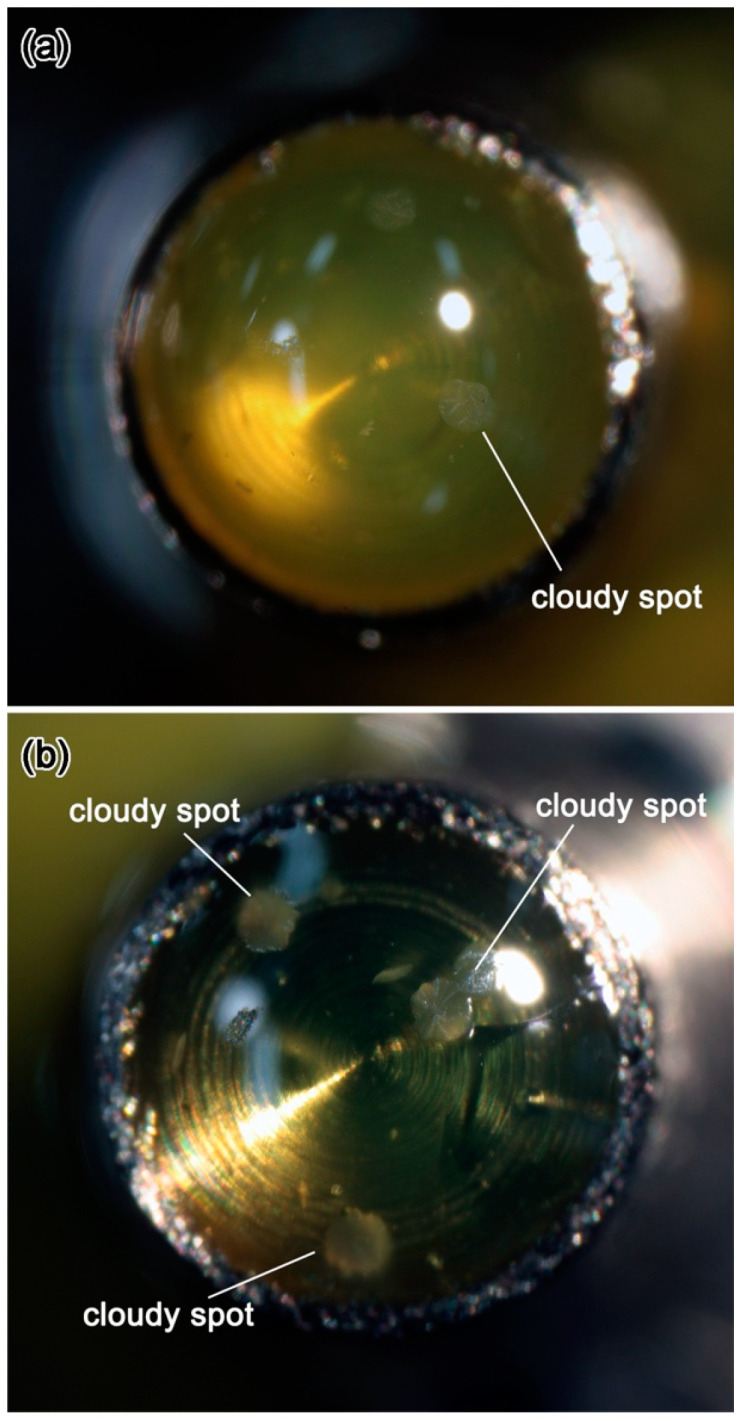
Photomicrographs of (**a**) slightly turbid and (**b**) clear transparent glassy sulfur droplets on the top face of stainless stubs. Ultrathin sections of embedded samples can be prepared by using glassy sulfur with transparency as shown in these photomicrographs. The diameters of these droplets are ~2 mm.

In the previous methods, small fragments of crystalline sulfur with a sample was melted on a glass slide to make a sulfur melt droplet containing a sample. After solidification of the droplet, it was removed from the glass slide and the droplet was attached on a stub by glue, e.g., [1,16]. In this protocol (Figure 1), we excluded these remove and attachment procedures. This protocol avoids thin cracks in a sulfur droplet due to mishandling, which may introduce organic contamination through thin cracks. In addition, in the protocol, translucent or even transparent sulfur droplets are obtained (Figure 2), which greatly improves visibility of fine-grained samples under microscopes during trimming of glassy sulfur and ultramicrotomy of tiny samples. Detailed protocols to obtain the sulfur droplets will be described later.

### 3.1. Stainless Stub

**Figure 3:**
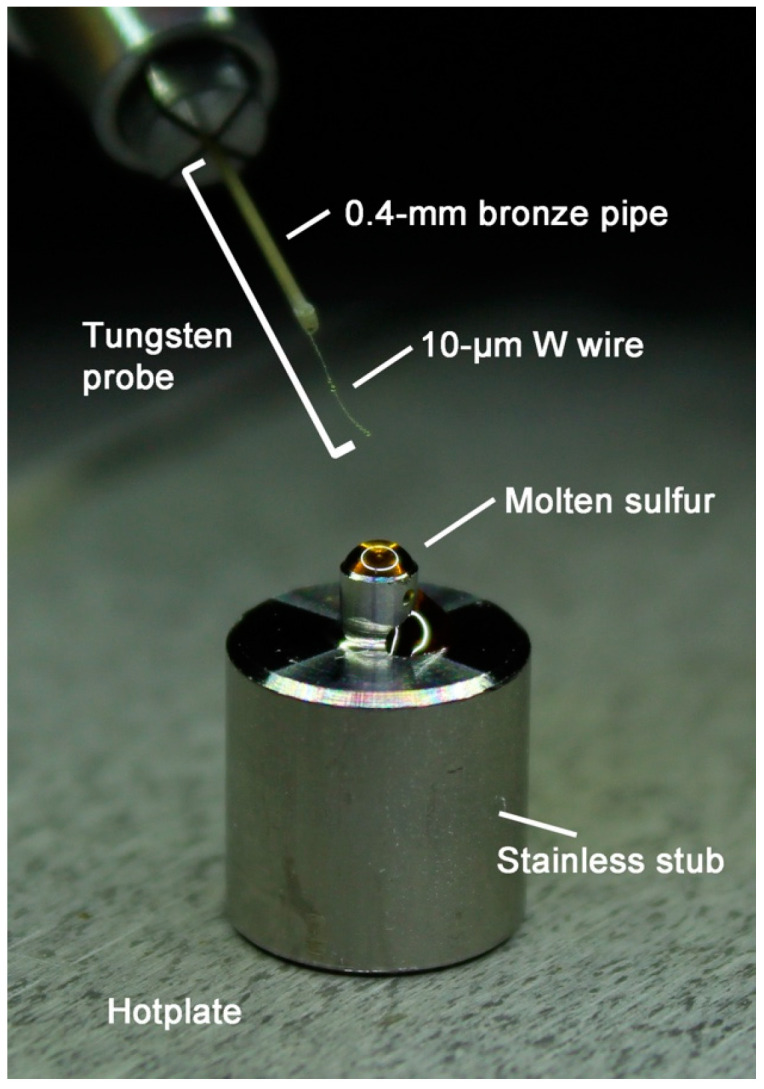
Stainless stub and a tungsten probe equipped on a micromanipulator. Image shows a suitable amount of a liquid sulfur droplet for safe ultramicrotomy.

To obtain clear sulfur droplets, stainless-steel stubs were specially designed for this new protocol (Figure 3). The lower part of the stub is 8 mm in diameter, which is common to typical epoxy stubs for ultramicrotomy. The upper part of the stub is 2 mm in diameter and 2 mm in height. Stubs made of the other material, such as heat-resistant glass, can certainly be used. We selected stainless steel because it can be processed easily by using a lathe to manufacture stubs. After making the stubs, machine oil should be carefully removed from them. The stubs should be cleaned by ultrasonication in acetone for 5 min. This process should be repeated 3 times; the stubs should be wiped well with cleaning tissues.

### 3.2. Use of Viscous Liquid Sulfur

Although some researchers recognize the usefulness of the viscous polymerized liquid sulfur, low-viscosity liquid sulfur has been used in the conventional, widely used protocols e.g., [14]. In our protocol, polymerized sulfur is used. Sulfur is a unique material that shows equilibrium polymerization in the liquid state [17,18,19,20]. The viscosity of liquid sulfur increases strikingly around 159.4 °C [19] that is known as λ temperature (T_λ_). Above T_λ_, as cyclic octa-atomic sulfur (S_8_) units polymerize, the viscosity of liquid sulfur increases [21]. Highly viscous liquid sulfur is needed to embed a sample because it serves to prohibit depolymerization of liquid sulfur during quenching. It also prevents for the sample from sinking to the bottom of the liquid sulfur droplet. Average S_8_ polymer chain length reaches the maximum value at ~170 °C [21]. The average polymer chain length is related to the viscosity of the liquid sulfur.

Because the color of liquid polymeric sulfur is dark yellow [19] and references therein, we are able to recognize polymerization by watching the color change of the droplet under a binocular stereo microscope. A compact hotplate is very useful to perform micromanipulation under a binocular stereomicroscope. In this protocol, the sulfur droplet is heated to ~170–180 °C based on the measurement by using a contact thermometer. Because polymerization rate of liquid sulfur is temperature dependent [21], it takes time to make a highly viscous liquid sulfur. In the following sections, we describe the details of this protocol according to the flow chart shown in Figure 1.

### 3.3. Details of the Protocol 1: Melting of Sulfur on a Hot Plate

**Figure 4:**
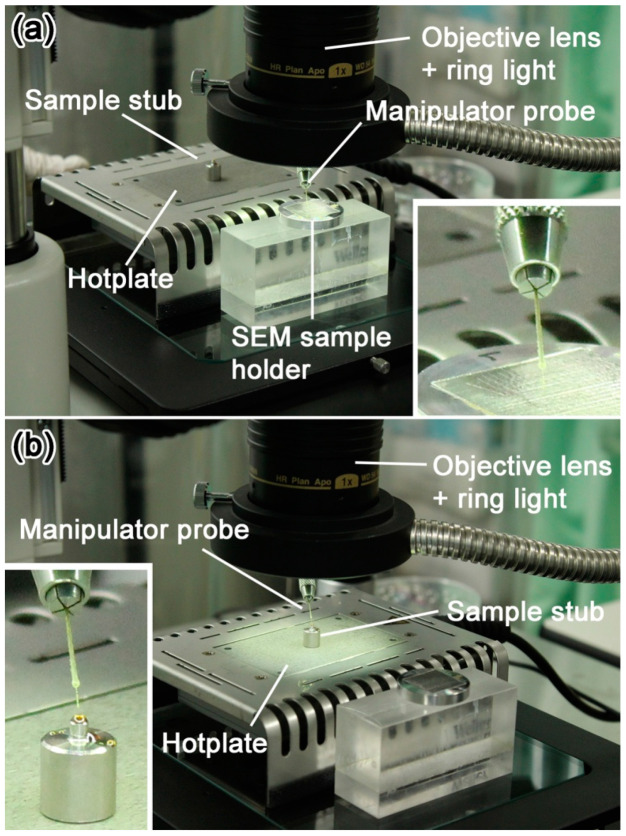
Sample embedding process. (**a**) Fine-grained sample on a SEM sample holder is picked up by using a tungsten probe under a stereomicroscope; (**b**) picked-up small sample is embedded in a liquid sulfur droplet under a stereomicroscope.

Sulfur powder (purity 99.99%) or a small fragment of sulfur crystal (purity 99.999%) is set on the top side by using a tiny medicine spoon or a pair of tweezers. Then the stub is carefully moved to a small hot plate that can be used under a stereomicroscope (Figure 4). Figure 2 shows glassy sulfur droplets with suitable sizes for ultramicrotomy viewed from the direction normal to the top side of the stubs. When the edge of a sulfur droplet is at the top face rim of the stub as shown in Figure 2, the height of the droplet is high enough to prevent a diamond knife from hitting against the stainless stub during ultramicrotomy. In case that the amount of sulfur becomes less, the stub must be removed from the hotplate and sulfur powder or fragment must be added so that the sulfur is melted again.

Because sulfur fumes are toxic for humans, we use a local ventilation equipment to decrease the aspiration of toxic sulfur fumes during melting of sulfur and embedding processes. The local ventilation equipment may also serve to reduce corrosion of glass microscope optics by sulfur fumes.

### 3.4. Details of the Protocol 2: Recognition of Polymerization of Liquid Sulfur

**Figure 5:**
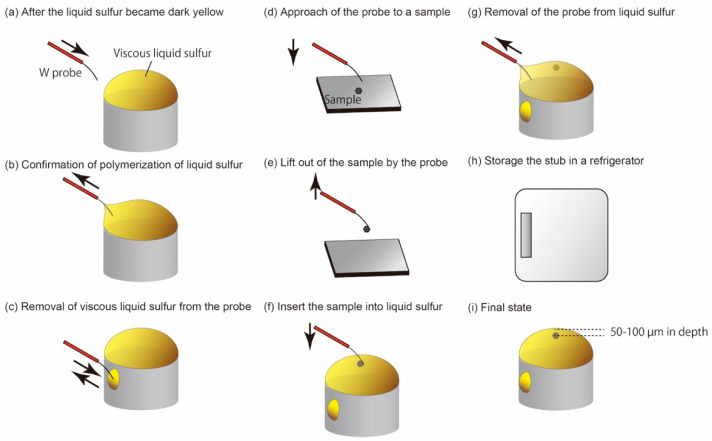
Conceptual diagrams of embedding processes in our protocol.

As described in Section 3.2, we use viscous polymerized liquid sulfur for embedding a small sample. In our case, when we set ~195 °C on the display of the controller of the hot plate, the top face of a stainless stub reaches to 170–180 °C. It was difficult to measure the exact temperature of the top face of the stubs by a contact thermometer because the instrument readings fluctuated from ~170 to ~180 °C. It takes 3–5 min to make a sulfur droplet viscous from sulfur powder. In contrast, it takes at least ~20 min to make a sulfur droplet viscous enough from sulfur crystal. However, when we remelt a glassy sulfur, which is once made by melting sulfur crystal, the sulfur droplet becomes viscous rapidly as is the case of sulfur droplets made from sulfur powder. After a liquid sulfur droplet becomes viscous, the droplet is stirred by a thin (10 µm in diameter) tungsten probe by using a micromanipulator. A ready-made thin tungsten probe can be also certainly used. Occasionally, tungsten probes may shed small opaque particles that can be confused for the embedded particle itself. You must keep the tungsten probe in the liquid sulfur under observation not to confuse them with the sample. After stirring, most liquid sulfur attached on the tungsten wire is removed except that a very small droplet of the liquid sulfur on the tip of the tungsten wire. The removed sulfur is attached to the cylindrical face of the upper part of the stainless stub (Figure 5c). Based on our trial, ~70 to ~80% of glassy sulfur droplets are kept mostly transparent till the next day. Ideally, however, ultrathin sections should be made on the same day.

### 3.5. Details of the Protocols 3–5: Picking-Up and Embedding of Fine-Grained Samples and Solidification of Liquid Sulfur

After the protocol 2, a small sample is picked up from a sample holder by using the tungsten probe under the stereomicroscope (Figure 5e). A manual XY stage equipped on a binocular stereomicroscope is useful to change the view field from the hot plate to the sample holder quickly. Because the liquid sulfur is so sticky that the tungsten probe should be withdrawn from the liquid sulfur (Figure 5g). The sample is embedded in the sulfur droplet under the stereomicroscope. The best depth of the embedded sample is ~50–100 µm from the top of the droplet. After embedding the sample, the stub is stored in a small refrigerator rapidly by using tweezers to quench the polymerized liquid sulfur. The stub is stored in the refrigerator for 20–30 min to solidify in a glassy state. Alternatively, the stub is set on a cold aluminum slab that was cooled to ~10 °C in the refrigerator after the stub was cooled in the refrigerator for 0.5–1 min. As a result, relatively clear glassy sulfur with low turbidity is obtained (Figure 2). In Figure 2, there are small cloudy spots on the surfaces of the glassy sulfur droplets. These spots are radial aggregates of acicular sulfur crystals. If the sample is not incorporated in such a spot, there is no problem to make ultrathin sections of the embedded sample. However, if the sample is unfortunately incorporated in such a spot, the stub must be heated again to melt the glassy sulfur and pick the sample out from the liquid sulfur by using a tungsten probe for doing over the protocol again.

### 3.6. Details of the Protocol 6: Trimming of Glassy Sulfur by Using a Diamond Knife

After solidification of sulfur, the stub is set on a chuck for samples embedded in cylindrical capsules. In this protocol, a diamond trimming knife with inclined edges is used for preparing well-continued ribbons of ultrathin sections. Alternatively, it takes a much longer time to finish trimming than scraping by freehand trimming. After setting, the top of the glassy sulfur droplet is removed by using the diamond trimming knife until the embedded sample can be easily recognized by using a binocular stereomicroscope equipped on the ultramicrotome. The cutting speed is 0.7 mm/s and the thickness during trimming is 0.5 µm.

**Figure 6:**
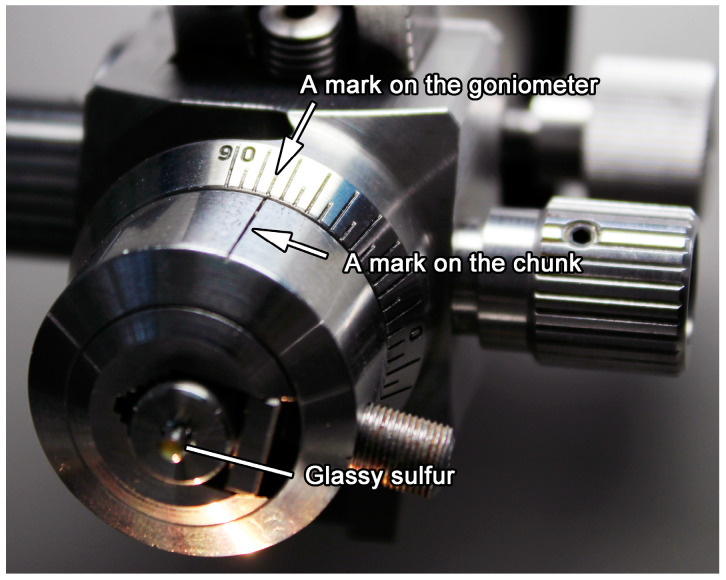
Marks on the chuck and the goniometer are indicated by arrows.

**Figure 7:**
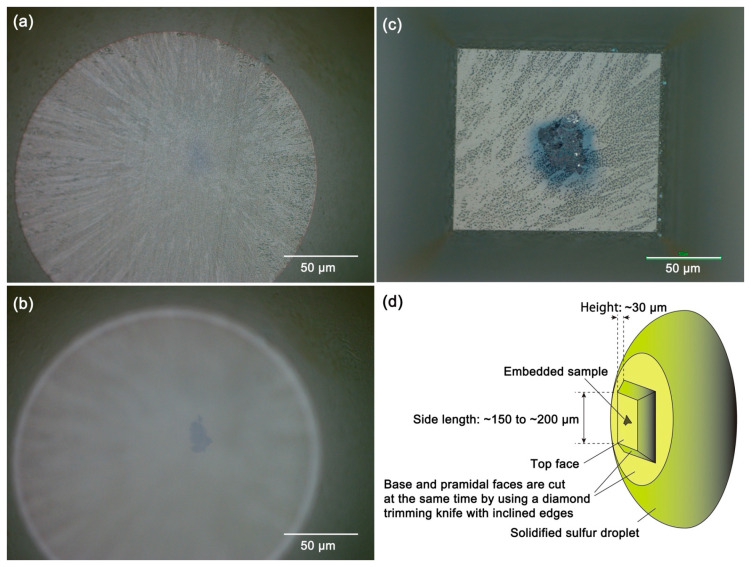
Photomicrographs of the embedded samples (**a**,**b**) during trimming, (**c**) after ultramicrotomy and (**d**) a schematic diagram of the shape of glassy sulfur on the top face of a stainless stub after trimming and ultramicrotomy. (**a**) The surface of sulfur comes into focus; (**b**) the embedded sample comes into focus; (**c**) After ultramicrotomy, the remnant of the embedded sample with a very flat surface can be observed. The rectangular area in this photograph is the top face of the truncated pyramid of glassy sulfur as shown in (**d**). The texture on its surface became dusty due to vaporization of sulfur because glassy sulfur evaporates even under a room temperature.

The chuck is removed from the goniometer of the ultramicrotome to measure the depth of the embedded sample from the surface of sulfur by using a microscope with scale marks of 1-µm intervals. It is important to record the angle between a marker on the chuck and a mark on the goniometer (Figure 6) because the chuck is rotated 90 degrees clockwise and anticlockwise in the later trimming processes. Because the refractive index of molten sulfur is 1.91–1.93 [22], the real depth of the embedded sample is almost twice the apparent (measured) depth. However, because it is difficult to see the top of the embedded sample as shown in Figure 7a,b, it is safe to remove the glassy sulfur by measuring the depth (apparent thickness) after the chuck is set on the ultramicrotome with the same configuration. After repeating this process a few times, the top of the embedded sample comes just below the surface (a few µm) of the embedding sulfur.

**Figure 8:**
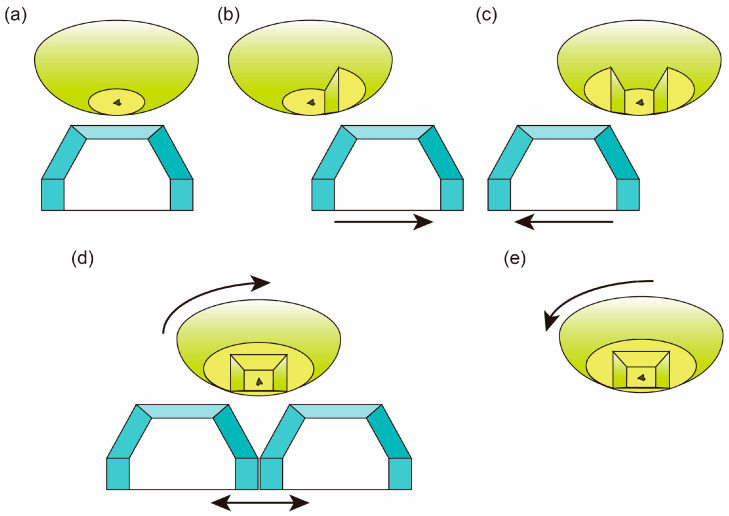
Conceptual diagrams of the trimming processes. (**a**) The top face is cut by using the horizontal edge of the trimming knife; (**b**,**c**) By using both horizontal and side edges, pyramidal edges and base faces are cut at the same time. Before cutting, the trimming knife is moved in the directions as shown in these figures; (**d**) Then the chuck is rotated 90 degrees clockwise and the other two sides and the base face are cut; (**e**) After trimming, the chuck is rotated 90 degrees anticlockwise.

Conceptual diagrams of the trimming processes are shown in Figure 8. After cutting the top face, the trimming knife is moved to cut one pyramidal side and the base face (Figure 8b). As shown in Figure 8b,c, one pyramidal side and the base face are cut at the same time. After two pyramidal sides and the base face were cut, the chuck is rotated 90 degrees clockwise and the other two pyramidal faces and the base face are cut (Figure 8d). Then, the chuck is rotated 90 degrees anticlockwise (Figure 8e). The sides of the top face are 150–200 µm in length for ~50 µm-sized samples (Figure 7c). The truncated pyramid is typically 30 µm in height (Figure 7d). The volatility of glassy sulfur is quite high even under a room temperature, its surface vaporizes quickly as shown in Figure 7c. Thus, it is important to move onto the next ultramicrotomy process immediately after trimming was finished.

### 3.7. Details of the Protocol 7: Cutting of Ultrathin Sections of Glassy Sulfur Embedding a Small Sample

**Figure 9:**
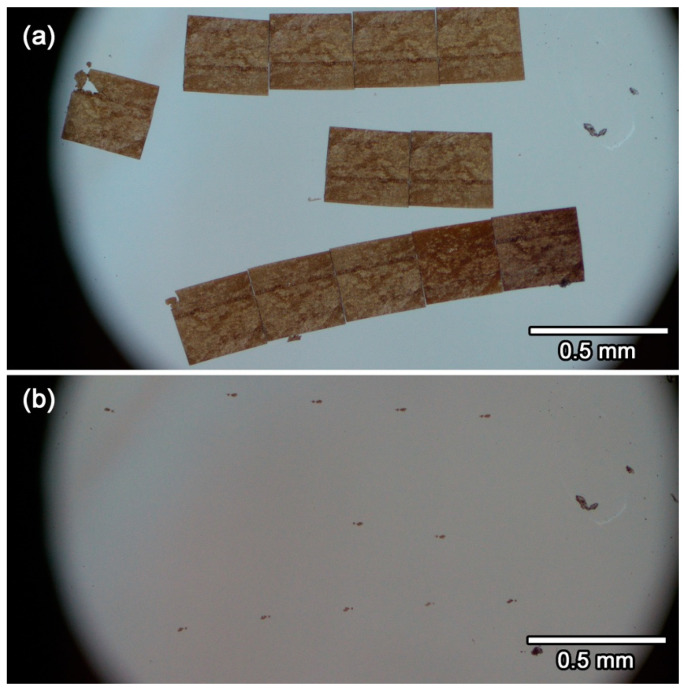
Ribbons of sulfur embedded ultrathin sections on a diamond plate (**a**) before and (**b**) after sublimation of sulfur. The arrays of thin foil samples in (**b**). Several particles whose positions are unchanged in both photomicrographs are contaminant grains attached during wiping the surface of the diamond plate.

**Figure 10:**
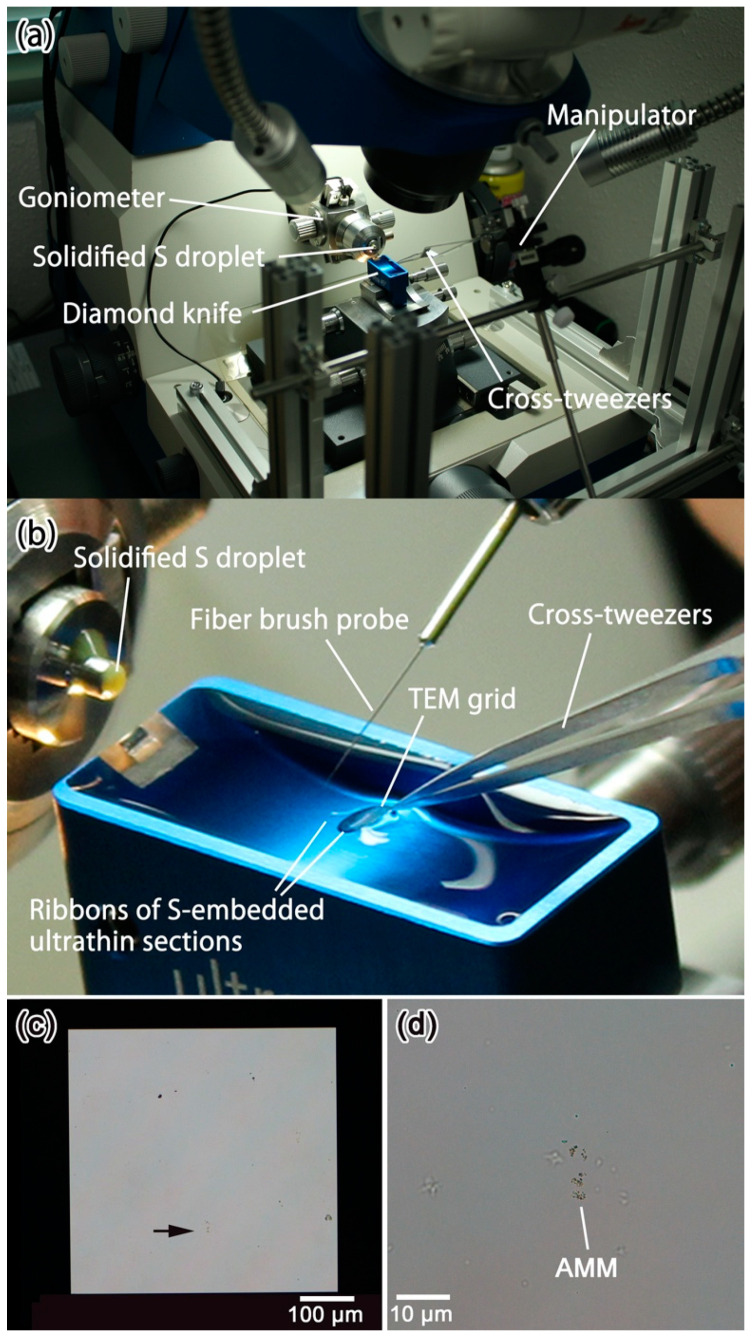
Tool to set a TEM grid in the trough water equipped on an ultramicrotome. (**a**) Whole view of the tool; (**b**) enlarged image shows how to control the position of a ribbon of ultrathin sections by using a fiber brush probe; (**c**) ultrathin sample of an Antarctic micrometeorite (AMM) on the silicon nitride window of a metallic Si TEM grid, indicated by an arrow; (**d**) enlarged image of the ultrathin sample of the AMM.

After adjusting the top face of the truncated pyramid parallel to the edge of a diamond knife for ultramicrotomy, ultrapure water is filled in the trough of the diamond knife. The amount of water is slightly more than that for ultramicrotomy of epoxy-embedded samples. Because ultrathin sections of sulfur are not drawn back during ultramicrotomy, slightly more water serves to decrease compression of sections. Well continued ribbons of ultrathin sections can be formed as shown in Figure 9a and Figure 10b.

### 3.8. Details of the Protocol 8–9: Scooping-Up of Ultrathin Section Ribbons of Glassy Sulfur Embedding a Small Sample, Followed by Evaporation of Glassy Sulfur

These ribbons can be easily scooped up by using a loop and the ribbons can be set on TEM grids as is the case in the conventional epoxy embedded ultrathin sections. This conventional scooping method is used to put ribbons onto optical grade ZnS crystal or diamond window for atomic force microscope—infrared spectroscopy (AFM–IR). Because these crystals are hydrophobic, a small strip of filter paper is attached on the edge of the loop to remove water quickly within the loop. The resultant ribbons on a diamond window is shown in Figure 9a. After the ribbons are air-dried, the diamond window is set in an isothermal bath at 80 °C more than 6 h to sublimate sulfur. After sublimation of sulfur, only ultrathin sections of the sample are left on the window (Figure 9b).

For STXM–XANES analysis, the ribbons are set on TEM grids with SiO_x_ supporting film or 500-µm-wide single-window metallic Si TEM grids with silicon nitride supporting film. The opening sizes of TEM grids with SiO_x_ supporting film are 300 mesh (~63 µm). Therefore, we made a tool to control the positions of ribbons on TEM grids in order to set as many ultramicrotomed sections as possible at the openings of these TEM grids (Figure 10). A cross tweezer, which is attached on a small manipulator, holds a TEM grid that is partially submerged in the trough water and controls its position (Figure 10a). After one or two ribbons of ultrathin sections were cut out of the sulfur embedded sample, the long side of the ribbons are attached on the waterfront line of the TEM grid carefully by using a cleaned fiber brush probe (Figure 10b). Then, the TEM grid is drawn back and recovered by using the manipulator. After removing water from the TEM grid by a filter paper, the grid is set on the filter paper in a petri dish. After the grid is air-dried, the grid is set in an isothermal bath at 80 °C more than 6 h to sublimate sulfur. Figure 10c shows a 500 µm-wide single window Si grid with silicon nitride supporting film. An arrow indicates that the position of an ultrathin section of an Antarctic micrometeorite (AMM) after sublimation of sulfur. Figure 10d shows an enlarged image of the ultrathin section of the AMM.

The obtained ultrathin foil samples are not only available for STXM–XANES and AFM–IR analyses, but also suitable for TEM observation and analysis if the ultrathin sections are on carbon supporting film. If TEM observation of ultrathin sections on SiO_x_ supporting film is required after STXM–XANES analysis, there are two ways to observe them by TEM; When one uses a TEM with a LB_6_ filament, there is no problem to observe them by the TEM because the supporting film will not be broken by charge-up due to low current density of LB_6_ filament. When one uses a field-emission TEM, the supporting film will be broken by charge-up unless the TEM grid is coated by carbon.

### 3.9. STXM–XANES Analysis

**Figure 11:**
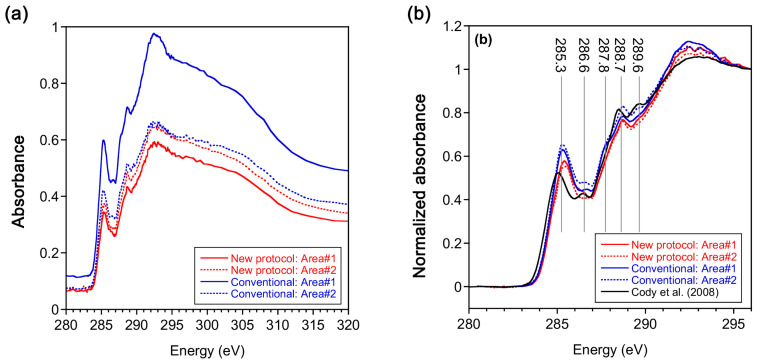
Carbon XANES spectra of Murchison insoluble organic matter (IOM) prepared by this protocol (“New protocol” in the caption) and by conventional protocol (“Conventional” in the caption). (**a**) Raw data obtained for two samples; (**b**) normalized and smoothed data for two samples. A carbon XANES spectrum of Murchison IOM prepared by the conventional protocol by [23] is also present in this figure for comparison.

**Figure 12:**
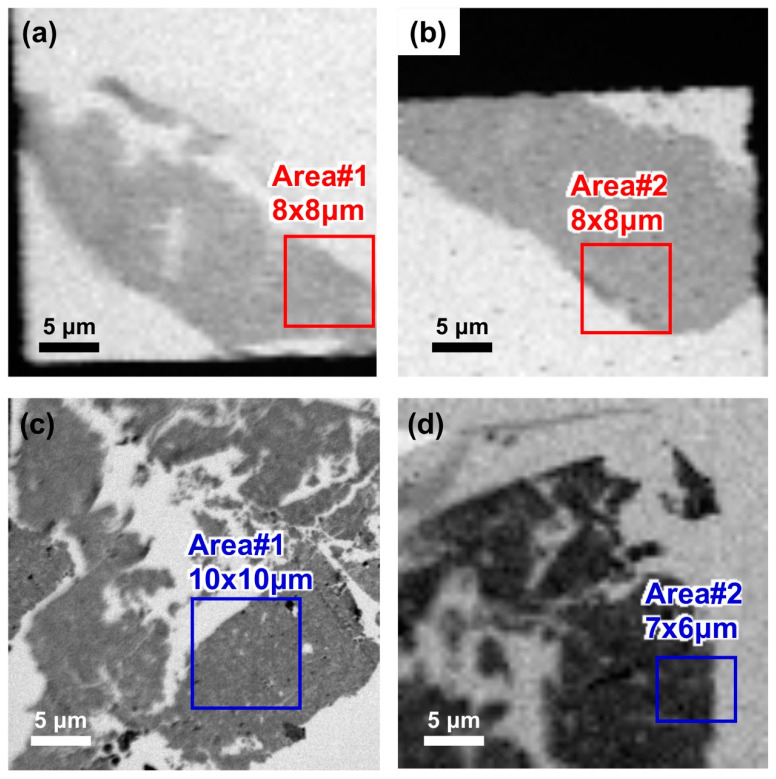
Carbon absorbance maps of the two samples measured in this study. (**a**,**b**) Thin foil samples prepared by the new protocol; (**c**,**d**) thin foil samples prepared by the conventional protocol. Analyzed areas are indicated by open squares in these images.

C-XANES spectra of the Murchison meteorite IOM sections prepared by this protocol and the conventional methods were compared (Figure 11 and Figure 12). All the C-XANES spectra show peaks at 285.3 eV assigned to aromatic C=C and 288.7 eV assigned to C(=O)O (carboxyl/ester), respectively. A small peak at 286.6 eV assigned to C=O and a shoulder at 287.8 eV assigned to aliphatic CH_x_ were also observed. The peak at 289.6 eV in one of the spectra obtained from the conventional method could be due to C-OH (hydroxyl). These features are consistent with C-XANES of Murchison IOM in literature which was also prepared by the conventional sulfur-embedding ultramicrotome method [23] (Figure 11b). Slight differences in peak intensities were observed between the samples prepared by the two different protocols, e.g., aromatic and aliphatic C were smaller in this protocol than conventional one. However, these differences could be attributed to local heterogeneity of IOM, considering that the C-XANES from [23] also show some differences.

## 4. Discussion and Conclusions

The difference between the two protocols is the temperatures of molten sulfur: 170–180 °C in the new protocol and ~150 °C or less in conventional one. If the temperature of new protocol affected the molecular structures of IOM, one may expect that the aromatic peak increased and aliphatic peak decreased, but it is not in the case. Calculation based on the kinetic experiments of decreases of aliphatic groups in Murchison IOM by FTIR indicated that 10% decrease in aliphatic C–H functional groups would require over one day (~10^5^ s) at 170 °C in an inert atmosphere [24], although it should be accelerated with the presence of oxygen. Thus, it is unlikely that slight differences in aliphatic features are due to the higher temperatures of molten sulfur.

In conclusion, another protocol to make sulfur embedded ultrathin sections was successfully developed for STXM–XANES, AFM–IR and TEM analyses. We use custom-made stubs made of stainless steel on which sulfur is melted, which reduces possible contamination. Polymerized liquid sulfur instead of low-viscosity liquid sulfur is the embedding media in this protocol. Due to high viscosity of the polymerized liquid sulfur, embedded samples stay near the surface of polymerized liquid sulfur, which facilitates trimming of glassy sulfur and ultramicrotomy of tiny samples. In addition, well-extended ribbons of ultramicrotomed thin sections can be obtained for STXM–XANES, AFM–IR and TEM analyses. By using a special tool to control the positions of the ribbons on TEM grids, we were able to improve the yield of ultrathin foil samples on the openings of these TEM grids. Because the Carbon XANES spectra of foil samples prepared by this protocol are consistent with those of the sections prepared by previous protocol, this protocol gives another alternative to prepare sulfur embedded ultramicrotomed sections. Therefore, it is expected that ultrathin samples prepared by this new protocol will enable the in situ analysis of prebiotic organic materials in the early Solar System, without modification of the returned asteroid samples.
